# Reperfusion measurements, treatment time, and outcomes in patients receiving endovascular treatment within 24 hours of last known well

**DOI:** 10.1111/cns.14080

**Published:** 2023-01-04

**Authors:** Lan Hong, Yifeng Ling, Yiran Zhang, Lumeng Yang, Siyuan Li, Xinyu Liu, Qiang Dong, Xin Cheng

**Affiliations:** ^1^ Department of Neurology, National Center for Neurological Disorders, National Clinical Research Centre for Aging and Medicine, Huashan Hospital, State Key Laboratory of Medical Neurobiology Fudan University Shanghai China

**Keywords:** acute stroke, endovascular treatment, outcome, reperfusion, treatment time

## Abstract

**Aims:**

The aim of this study was to explore the interaction between reperfusion and treatment time on the outcomes of patients undergoing endovascular treatment presenting within 24 h of last known well, and to compare the predictive ability of different reperfusion measurements on outcomes.

**Methods:**

Eligible patients from a single‐center cohort were enrolled in this study. Reperfusion was assessed using reperfusion index (decreased volume of hypoperfusion lesion compared with baseline) measured by repeated perfusion imaging, and modified treatment in cerebral ischemia score measured by digital subtraction angiography, respectively. The interactions between reperfusion measurements and treatment time on outcomes were explored using multivariate‐adjusted logistic and linear regression models. The predictive abilities of reperfusion measurements on outcomes were compared using area under the receiver operating characteristic curve (ROC‐AUC) and values of R‐square.

**Results:**

Reperfusion index and treatment time had significant interactions on 3‐month modified Rankin Scale (mRS) 0–2 and infarct growth (*p* for interaction <0.05). Although the AUCs were statistically similar (AUCs of mRS 0–2 prediction, mTICI≥2b:0.63, mTICI≥2c:0.59, reperfusion index≥0.5:0.66, reperfusion index ≥0.9:0.73, P value of any of the two AUCs >0.05), reperfusion index≥0.9 showed the highest R‐square values in outcome prediction (R‐square values of 3‐month mRS 0–2 and infarct growth = 0.21) among all the reperfusion measurements.

**Conclusion:**

Treatment time mitigated the effect of reperfusion on outcomes of patients receiving endovascular treatment within 24 h of last known well. Reperfusion index≥0.9 might serve as a better proxy of good outcomes compared with other reperfusion measurements.

## Graphical abstract

For acute stroke patients undergoing endovascular treatment within 24 hours of last known well, delayed treatment time could still mitigate the benefit of endovascular treatment, even under the selection of advanced neuroimaging. Additionally, a posttreatment perfusion scan may be worthwhile to re‐assess their perfusion condition, in case intervention is needed to improve clinical outcomes.

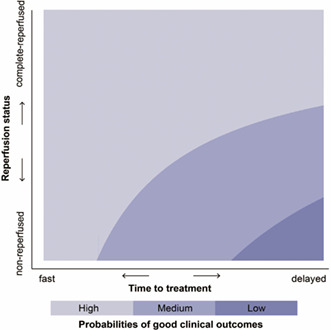

## 1 INTRODUCTION

Emergent endovascular treatment (EVT) has been a routine practice for acute large vessel occlusion (LVO) patients presenting within 24 hours of last known well (LKN).^1^, ^2^ Reperfusion is the key determinant of EVT efficacy, and is time‐dependent with greater benefit of earlier treatment in patients punctured within 12 h of LKN.^3^, ^4^, ^5^, ^6^, ^7^, ^8^ However, a post hoc analysis of the Diffusion and Perfusion Imaging Evaluation for Understanding Stroke Evolution 2 (DEFUSE 2) study has shown that the benefit of EVT would not be undermined by delayed time in patients with imaging selection,^9^ where the number of patients with good collaterals, also known as the “slow progressors” increases with time.^3^, ^10^ Whereas, there are odds where collateral flow can fail over time even for patients showing benign perfusion profiles.^11^ Additionally, the stringent target mismatch criteria in DEFUSE serious studies^12^, ^13^ combining mismatch ratio, volume of core, penumbra, and areas with severe delay are hard to strictly follow under real‐world clinical practice, with which a considerable number of patients will be ruled out. Therefore, we hypothesized that reperfusion and treatment time had interactions on outcomes in patients undergoing EVT within 24 h of LKN in real‐world practice.

Reperfusion restoration measured by repeated perfusion imaging and modified treatment in cerebral ischemia (mTICI) score measured by digital subtraction angiography (DSA) imaging are the two major measurements of reperfusion status post‐EVT.^12^, ^14^, ^15^ Nevertheless, few studies directly compare the predictive ability of these two measurements in outcome prediction.^16^

Imaging and clinical analysis of real‐world EVT patient data who follow a more flexible mismatch standard of imaging selection can provide a unique prospective of associations between treatment time and outcomes, as well as the prediction ability of different reperfusion measurements. Therefore, using the data of a single‐center cohort of EVT patients who presented within 24 h of LKN, the aim of this study was to (1) explore the interaction between the various treatment time metrics and reperfusion on different outcomes and (2) to compare the predictive performance of different reperfusion measurements obtained by repeated perfusion imaging and DSA on outcomes.

## 2 METHODS

### 2.1 Patient selection

Consecutive acute ischemic stroke patients within 24 h of LKN admitted to the Department of Neurology, Huashan Hospital from April 2015 to February 2021 were prospectively registered. Patients meeting the following criteria were retrospectively enrolled in this study: (1) underwent complete baseline multimodal computed tomography (CT) imaging, including noncontrast CT (NCCT), CT perfusion (CTP), CT angiography (CTA); (2) had large vessel occlusion/ severe stenosis of anterior cerebral circulation (Severe stenosis was defined as >50% stenosis of the vessel caliber in CTA referred to the diameter of the ipsilateral adjacent contact segment); (3) had complete baseline clinical profiles and 3‐month follow‐up; (4) underwent emergent EVT; (5) had repeated CTP scan within 24–48 h post‐EVT and follow‐up noncontrast CT or diffusion‐weighted imaging (DWI) within 7 days post‐EVT. Written informed consent was obtained from each participant for data collection, analysis, and publishment. The study was approved by the ethics committee of Huashan Hospital, Fudan University. The primary outcome of this study was 3‐month good functional outcome, defined as a modified Rankin scale (mRS) of 0–2. The secondary outcome was infarct growth.

All of the AIS patients within 24 h of LKN arriving at the emergency room (ER) of Huashan Hospital routinely underwent multimodal imaging scan for perfusion and angiographic evaluation if the patients had no contraindication for contrast agent injection. Before April 2018, only patients presenting within 6 h from LKN were considered eligible for EVT, after which the time window was extended to 24 h. All the perfusion images were real‐time postprocessed by MIStar (Apollo Medical Imaging Technology) with single value deconvolution with delay and dispersion correction. Only patients with a mismatch ratio (volume of hypoperfused leison /core volume) > 1.2 were considered candidates for EVT. For patients with a baseline core volume larger than 70 ml, whether they were eligible for EVT was depended on the comprehensive judgment of the stroke neurologists and neurointerventionists taking the demographic and clinical characteristics into consideration. The following time points were recorded for each patient: time of LKN, time of arrival at the ER, time of baseline multimodal imaging, time of groin puncture, and time of reperfusion (for patients who achieved at least partial reperfusion). The following time intervals were calculated for the interactions between reperfusion measurements: Time from LKN to ER arrival, time from LKN to acute multimodal imaging, time from LKN to groin puncture and time from LKN to reperfusion. Patients who arrived within 4.5 h of LKN were thrombolyzed with intravenous rtPA according to the latest Chinese guidelines of management of acute ischemic stroke. Patients underwent 24–48‐hour NCCT and CTP scan post‐EVT if there was no contraindication.

### 2.2 Imaging protocol and imaging analysis

For baseline and 24–48‐hour multimodal CT scan, a 64‐slice detector scanner (Discovery CT750 HD; GE Medical Systems) was used. The scanning parameters were as follows: Jog mode, 80 kVp/220 mAs; 26 cycles for 42 s; 312 slices. A dual‐head power injector (Stellant Injection System; Medrad Inc., Indianola) was used to inject 40 ml of a nonionic contrast medium (Ultravist, iodine 370 mg I/mL; Bayer Healthcare) at 4.5 ml/s, followed by 20 ml saline. CTP scan was initiated at 7 s after the contrast agent bolus. CTA with acquisition from aorta arc to vertex was performed immediately after perfusion CT. Brain standard reconstruction was then performed. To avoid radiation exposure to the lens, the gantry angle was parallel to and above the orbital roof. Whether CTP and CTA were acquired before or after intravenous rtPA depended on the judgment of stroke neurologists.

All patients underwent follow‐up magnetic resonance imaging (MRI) scan within 7 days after stroke onset on a 3.0 T MRI scanner (Magnetom Verio; Siemens Healthcare) if there was no contraindication. The MRI imaging protocol included DWI and Fluid‐Attenuation Inversion Recovery (FLAIR) imaging. Otherwise, head NCCT was conducted to assess the infarct volume.

All of the perfusion images were centrally re‐analyzed using MIStar (Apollo Medical Imaging Technology, Melbourne) with single value deconvolution with delay and dispersion correction by a neurologist (Dr. Lan Hong) who was blinded to the analysis of DSA imaging and the clinical outcome of the patients when analyzing perfusion imaging. Hypoperfusion volume and core volume were calculated using previously validated thresholds (Hypoperfusion lesion: delay time [DT] > 3 s, Core: relative cerebral blood flow[rCBF] < 30%).^17^ Reperfusion index was calculated using the following equation: (Hypoperfused lesion_baselineCTP_ – Hypoperfused lesion_24‐48hCTP_)/ Hypoperfused lesion_baselineCTP_. Reperfusion index≥0.5 was considered as major reperfusion,^12^ and reperfusion index≥0.9 was considered as complete reperfusion.^18^ The final infarct volume (FIV) was also calculated using MIStar with planimetric techniques by semi‐automatically drawing regions of interests (ROIs) in DWI/NCCT images. Infarct growth was calculated by subtracting baseline core volume from the final infarct volume. Imaging of DSA was centrally reviewed by a neurointerventionist (Dr. YF Ling) with 2‐year experience of neurointervention who was blinded to the reperfusion analysis and clinical outcome. Partial reperfusion was considered as mTICI score ≥ 2a. Major reperfusion was defined as mTICI score ≥ 2b, and complete reperfusion was defined as mTICI score ≥ 2c.^15^

### 2.3 Statistical analysis

Statistical analysis was performed using Stata v15.1 (StataCorp, College Station). Graphs were drawn using Stata v15.1 (StataCorp, College Station) and Prism v8.0 (GraphPad Software). A two‐tailed *p* < 0.05 was considered as significant. Mean and standard deviation were used to describe continuous variables if normally distributed, or median and interquartile range (IQR) if skewedly distributed. Categorical variables were described using percentage. For continuous variables, normality was tested using Shapiro–Wilk test. Differences of demographic and clinical and imaging data were compared using Student's *t*‐test, Wilcoxon rank‐sum test or Kruskal–Wallis test for continuous variables, and χ2 test or Fisher's exact test for categorical variables. The correlation between any two continuous variables was tested using Spearman correlation coefficient if not normally distributed, or Pearson correlation coefficient if normally distributed. The interaction between reperfusion index / mTICI score and each time interval was tested using multivariate‐adjusted linear/logistic regression model adjusted by any baseline demographic, imaging and clinical variables that were significant in the univariate analysis or variables that were considered clinically relevant.

The predictive ability of reperfusion measurements, including major/complete reperfusion assessed by CTP/DSA on the functional outcome was calculated using the area under the receiver operating characteristic curve (AUC‐ROC) and compared using a method developed by Pepe et al. and Janes et al.^19^, ^20^ adjusted for other baseline demographic, imaging and clinical variables that were significant in the univariate analysis or variables that were considered clinically relevant. Additionally, R‐square values were also calculated using multivariate logistic regression models. The one with the highest AUC and R‐square value was considered the best predictor of functional independency. For infarct growth, multivariate simple linear regression models were used to assess reperfusion measurements in infarct growth prediction. R‐square values of the linear regression models were also provided to compare their predictive value.

In this study, any multivariate analysis concerning mRS 0–2 were adjusted by age and baseline National Institutes of Health Stroke Scale (NIHSS) score as statistically significant or clinically relevant baseline variables, and any multivariate analysis concerning infarct growth were adjusted by baseline core volume, history of diabetes mellitus and MR/NCCT scan as statistically significant or clinically relevant variables.

## 3 RESULTS

From April 2015 to February 2021, a total number of 870 patients with 24‐hour of LKN were admitted to the Department of Neurology, Huashan Hospital. Among them, 194 patients underwent emergent EVT. After excluding 80 patients with incomplete clinical or imaging data and 41 patients with LVO of posterior circulation, a number of 73 patients were included in this study. The mean (SD) age was 66.5 (13.3) years old, with mean (SD) baseline NIHSS of 13.7 (5.0). The details of baseline and follow‐up demographic, imaging and clinical outcomes were listed in Table 1.

**TABLE 1:** Baseline and follow‐up clinical and imaging data of the whole cohort^a^.

	*n* = 73
Baseline Demographics	
Age, median (IQR), yrs	66.5 (13.3)
Male	44 (60.3%)
Baseline Glucose, median (IQR), mmol/L	7.0 (6.2, 8.1)
Baseline SBP, mean (SD), mmHg (*n* = 92)	142.4 (22.3)
Baseline DBP, mean (SD), mmHg (*n* = 92)	84.1 (12.4)
Baseline NIHSS, mean (SD)	13.7 (5.0)
Medical History	
History of smoking	27 (37.0%)
History of hypertension	39 (53.4%)
History of atrial fibrillation	22 (30.1%)
History of dyslipidemia	9 (12.3%)
History of diabetes mellitus	14 (19.2%)
Past history of stroke or TIA	14 (19.2%)
History of congestive heart failure	1 (1.4%)
History of ischemic heart disease	4 (5.5%)
Taking antiplatelet prior to stroke	13 (17.8%)
Taking anticoagulant prior to stroke	8 (11.0%)
Cause of Stroke	
Large artery atherosclerosis	37 (50.7%)
Cardiac embolism	25 (34.3%)
Others^b^	11 (15.0%)
Occlusion Site	
ICA	17 (23.3%)
MCA‐M1	45 (61.6%)
MCA‐M2	6 (8.2%)
Tandem	5 (6.9%)
Reperfusion Treatment Procedure	
Procedure Time	
Time from LKN to ER arrival, median (IQR), min	170.0 (85.5, 309.5)
Time from LKN to acute multimodal imaging, median (IQR), min	229.0 (122.0, 362.0)
Time from LKN to groin puncture, median (IQR), min	350.0 (230.0, 539.0)
Time from LKN to reperfusion, median (IQR), min^c^	430.0 (325.0, 561.0)
Intravenous thrombolysis	30 (41.1%)
Final mTICI	
0	10 (13.7%)
1	4 (5.5%)
2a	8 (11.0%)
2b	15 (20.6%)
2c	6 (8.2%)
3	30 (41.1%)
Perfusion Imaging data	
Baseline infarct core, median (IQR), ml	10.0 (4.0, 21.0)
Baseline DT >3 s, median (IQR), ml	88.0 (52.5,144.5)
Reperfusion index, median (IQR)	1.0 (0.6, 1.0)
Reperfusion index≥0.5	58 (79.5%)
Reperfusion index≥0.9	40 (54.8%)
Outcomes	
Infarct growth, median (IQR), ml^d^	15.5 (2.2, 61.6)
3 m mRS 0–2	34 (46.6%)

Abbreviations: DBP, diastolic blood pressure; DT, Delay time; mRS modified Rankin Scale; ICA, internal carotid artery; IQR, Interquartile range; LKN, Last known well; ER Emergency room; MCA‐M1 M1, segment of middle cerebral artery; MCA‐M2 M2, segment of middle cerebral artery; mTICI, modified Treatment in Cerebral Ischemia; NCCT, Non‐contrast computed tomography; NIHSS, National Institutes of Health Stroke Scale; SBP, systolic blood pressure; SD, Standard deviation; TIA, transient ischemic attack.

^a^ Data are presented as number (percentage) of patients unless otherwise indicated.

^b^ Other causes of stroke included embolic stroke of undetermined source, hypercoagulation, stroke of undermined causes, dissection of ipsilateral carotid artery, syphilis and hypoperfusion.

^c^ Only calculated for patients who had final mTICI≥2a, *n* = 59.

^d^ Final infarct volume of 18 Patients were measured using NCCT.

### 3.1 Interaction between time intervals and major/complete reperfusion on outcomes

Among all the baseline demographic, clinical and imaging data, only age was significantly different between patients with 3‐month mRS 0–2 and patients without 3‐month mRS 0–2 in the univariate analysis. For treatment procedure metrics, more patients with 3‐month mRS 0–2 achieved mTICI≥2b on the final angiographic run of DSA imaging. For the post‐EVT metrics, patients with 3‐month mRS 0–2 showed smaller infarct core volume and smaller volume of hypoperfusion lesion on the 24‐ to 48‐hour perfusion CT, smaller final infarct volume on the follow‐up DWI/NCCT imaging scan, and a higher rate of reperfusion index≥0.5 as well as reperfusion index≥0.9 (Table S1). Therefore, age, together with baseline NIHSS, was entered in the multivariate‐adjusted models of 3‐month mRS 0–2 as statistically significant or clinically relevant baseline variables. As for the univariate analysis concerning infarct growth, only history of diabetes mellitus and baseline infarct core showed trend of significance among all the baseline variables (history of diabetes mellitus *p* = 0.06, baseline infarct core *p* = 0.05, Table S2). Therefore, baseline core volume, history of diabetes mellitus, and MR/NCCT scan were entered in the multivariate‐adjusted model of infarct growth as statistically significant or clinically relevant baseline variables.

**FIGURE 1:**
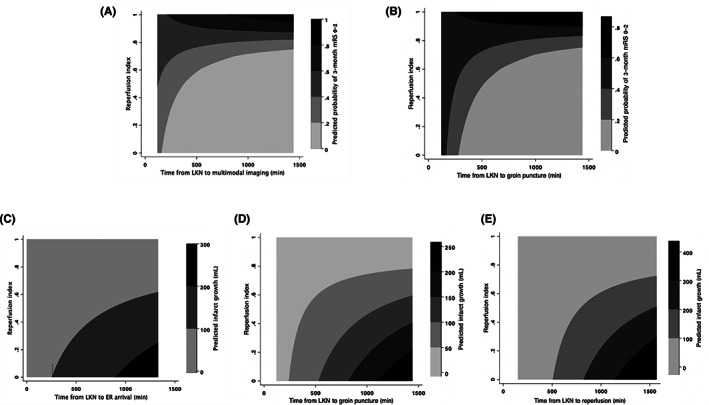
Interaction plots of reperfusion index and different treatment time intervals on outcomes (A). Interaction plot of reperfusion index and time from LKN to acute multimodal imaging on 3‐month mRS 0–2. (B). Interaction plot of reperfusion index and time from LKN to groin puncture on 3‐month mRS 0–2. (C). Interaction plot of reperfusion index and time from LKN to ER arrival on infarct growth. (D). Interaction plot of reperfusion index and time from LKN to groin puncture on infarct growth. (E). Interaction plot of reperfusion index and time from LKN to reperfusion on infarct growth. ER, emergency room; LKN, Last known well; mRS, modified Rankin Scale.

For all the time intervals, the interaction between time from LKN to groin puncture, time from LKN to multimodal imaging and reperfusion index on 3‐month mRS 0–2 was statistically significant, respectively (adjusted for age and baseline NIHSS, Time from LKN to multimodal imaging: *p* for interaction = 0.046, Figure 1A; time from LKN to groin puncture: *p* for interaction =0.035, Figure 1B). As for the prediction of infarct growth, the interaction between time from LKN to groin puncture, time from LKN to ER arrival, time from LKN to reperfusion and reperfusion index were statistically significant (adjusted for baseline core volume, history of diabetes mellitus and MR/NCCT scan, Time from LKN to ER arrival: P for interaction =0.04, Figure 1C; time from LKN to groin puncture: *p* for interaction = 0.02, Figure 1D; time from LKN to reperfusion: *p* for interaction =0.003, Figure 1E). No statistical significance was found between any time interval and mTICI score (Table S3).

### 3.2 Comparisons of the predictive ability of major/complete reperfusion of outcomes

**FIGURE 2:**
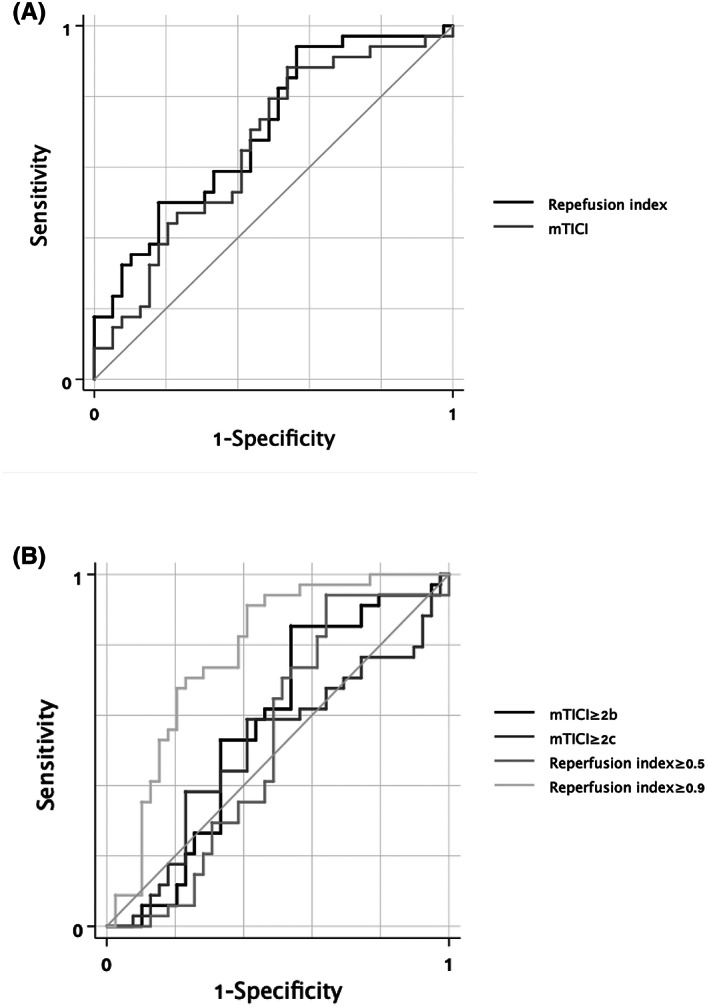
ROC‐AUC of different reperfusion measurements on 3‐month mRS 0–2 prediction, adjusted by age and baseline NIHSS (A). ROC‐AUC of reperfusion index and mTICI score, adjusted for age and baseline NIHSS. AUC of reperfusion index 0.71, 95% CI 0.58–0.83; AUC of mTICI score 0.66, 95% CI 0.52–0.80. *p* = 0.63. (B). ROC‐AUC of reperfusion index≥0.5, reperfusion index≥0.9, mTICI≥2b and mTICI≥2c, adjusted for age and baseline NIHSS. AUC of mTICI≥2b 0.63, 95% CI 0.50–0.77; AUC of mTICI≥2c 0.59, 95% CI 0.44–0.73; AUC of reperfusion index≥0.5 0.66, 95%CI 0.51–0.80; AUC of reperfusion index ≥0.9 0.73, 95%CI 0.58–0.87. AUC comparison: Reperfusion index≥0.5 vs. mTICI≥2b *p* = 0.76; reperfusion index≥0.5 vs. mTICI≥2c *p* = 0.49; mTICI≥2b vs. mTICI≥2c *p* = 0.51; reperfusion index≥0.9 vs. mTICI≥2b *p* = 0.33; reperfusion index≥0.9 vs. mTICI≥2c *p* = 0.25; reperfusion index≥0.9 vs. reperfusion index≥0.5 *p* = 0.42. CI, confidence interval; mRS, modified Rankin Scale; mTICI, modified treatment in cerebral ischemia; NIHSS, National Institutes of Health Stroke Scale; ROC‐AUC, area under the receiver operating characteristic curve.

**FIGURE 3:**
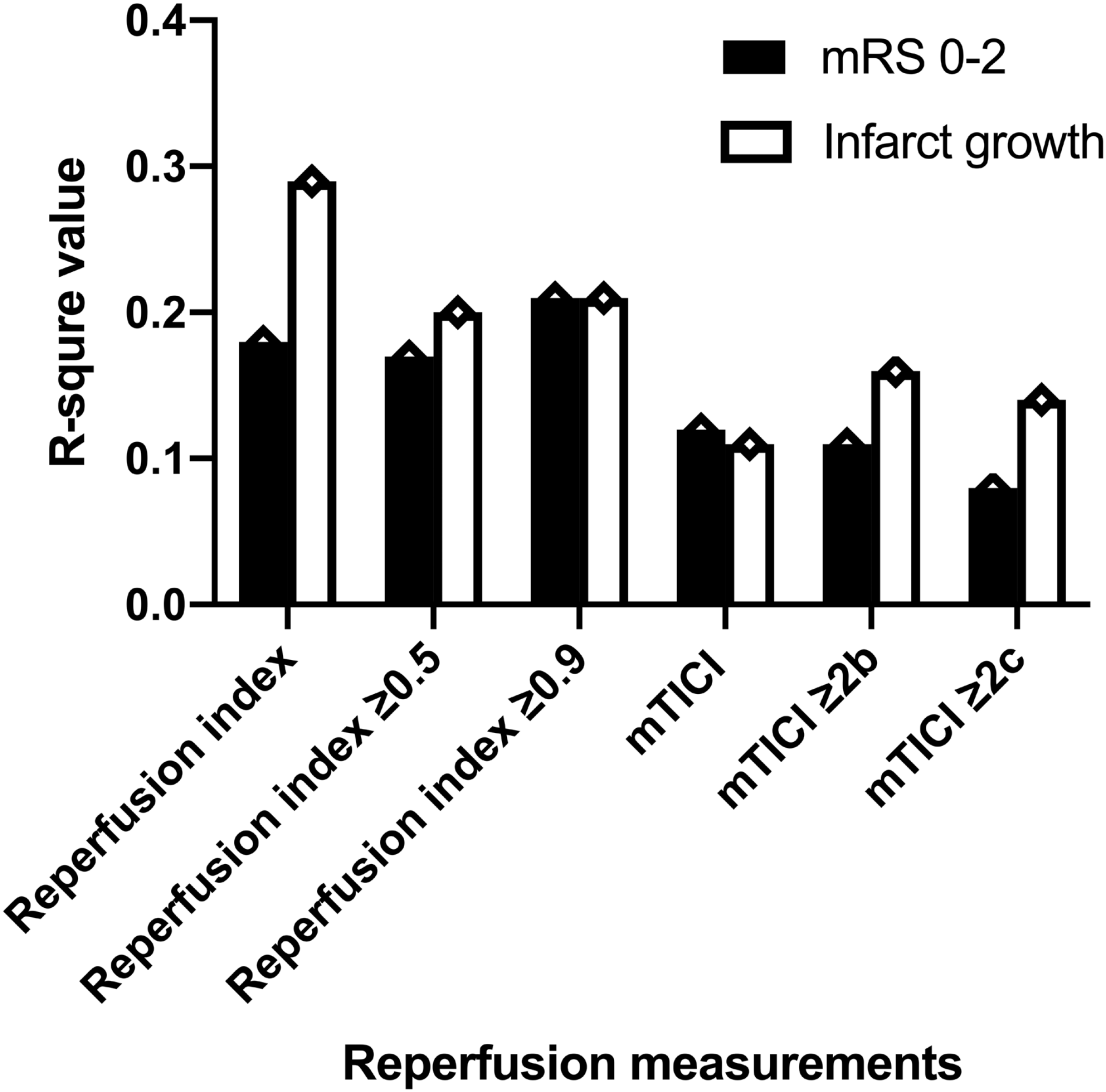
R‐square value of different reperfusion measurements on outcome prediction Prediction of 3‐month mRS 0–2: with age and baseline NIHSS entering the multivariate‐adjusted logistic regression model. Reperfusion index: 0.18; mTICI score 0.12; mTICI≥2b: 0.11; mTICI≥2c:0.08; reperfusion index≥0.5: 0.17; reperfusion index≥0.9: 0.21. Prediction of infarct growth: with history of diabetes mellitus, baseline core volume and MR/NCCT scan entering the multivariate‐adjusted linear regression model. Reperfusion index: 0.29; mTICI score 0.11; mTICI≥2b: 0.16; mTICI≥2c: 0.14; reperfusion index≥0.5: 0.20; reperfusion index≥0.9: 0.21. mRS, modified Rankin Scale; mTICI, modified treatment in cerebral ischemia; NIHSS, National Institutes of Health Stroke Scale.

The AUCs of reperfusion index was not statistically different from that of mTICI score (adjusted for age and baseline NIHSS, AUC of reperfusion index 0.71, 95% CI 0.58–0.83; AUC of mTICI score 0.66, 95% CI 0.52–0.80, *p* = 0.63, Figure 2A). Reperfusion index had a higher R‐square value in prediction of 3‐month mRS 0–2 (with age and baseline NIHSS entering the model as statically or clinically significant variables) and infarct growth (with history of diabetes mellitus, baseline core volume and MR/NCCT scan) than mTICI score (Reperfusion index vs mTICI score: mRS 0–2 0.18 vs. 0.12, infarct growth: 0.29 vs. 0.11, Figure 3).

The AUCs (95%CI) of mTICI≥2b, mTICI≥2c, reperfusion index≥0.5 and reperfusion index ≥0.9 on 3‐month mRS 0–2 were 0.63 (0.50, 0.77), 0.59 (0.44, 0.73), 0.66 (0.51, 0.80) and 0.73 (0.58, 0.87), respectively, with the adjustment of age and baseline NIHSS (Figure 2B). Although the AUC of reperfusion index ≥0.9 was numerically larger, no significant difference existed between any two AUCs (reperfusion index≥0.5 vs. mTICI≥2b *p* = 0.76; reperfusion index≥0.5 vs. mTICI≥2c *p* = 0.49; mTICI≥2b vs. mTICI≥2c *p* = 0.51; reperfusion index≥0.9 vs. mTICI≥2b *p* = 0.33; reperfusion index≥0.9 vs. mTICI≥2c *p* = 0.25; reperfusion index≥0.9 vs. reperfusion index≥0.5 *p* = 0.42). Reperfusion index≥0.9 had the highest R‐square value among all the reperfusion measurements for the prediction of 3‐month mRS 0–2, with age and baseline NIHSS entering the model (mTICI≥2b: 0.11; mTICI≥2c: 0.08; reperfusion index≥0.5: 0.17; reperfusion index≥0.9: 0.21, Figure 3). For the prediction of infarct growth, reperfusion index≥0.9 also showed the highest R‐square value among all the reperfusion measurements with history of diabetes mellitus, baseline core volume and MR/NCCT scan (mTICI≥2b: 0.16; mTICI≥2c:0.14; reperfusion index≥0.5: 0.20; reperfusion index≥0.9: 0.21, Figure 3).

## 4 DISCUSSION

This retrospective study with a single‐center EVT cohort of patients within 24 h of LKN has demonstrated that: (1) Reperfusion index and treatment time metrics had an interaction on 3‐month mRS 0–2 and infarct growth; (2) Reperfusion index≥0.9 might serve as a better proxy of better outcomes compared with reperfusion index≥0.5, mTICI≥2b and mTICI≥2c.

To our knowledge, this is the first study exploring the interaction of time and reperfusion status in acute ischemic stroke patients within 24 h from last known well in real‐world practice under the selection of advanced imaging. The findings of the modifying effect of time on the association between reperfusion and outcomes of EVT patients are in accordance with many other studies of large randomized controlled trials and observational registries.^3^, ^5^, ^7^ For patients with substantial reperfusion, delayed reperfusion is associated with decreased odds of functional independency,^7^, ^8^, ^10^, ^21^ which can be partly explained by infarct growth and reperfusion hemorrhage.^3^, ^5^ Get With the Guidelines‐Stroke (GWTG‐Stroke) registry has revealed a nonlinear association between benefit reduction and onset‐to‐puncture time, with a more prominent reduction in the 4.5 h and a slower decline in the 4.5–8 h after stroke onset. The application of imaging selection for EVT patients beyond traditional time windows allows the discrimination between the ones with poor collateral flow (the fast progressors) and the ones with good collateral flow (the slow progressors), and more of the latter ones are considered suitable for emergent EVT. It has been proven that the level of collateral flow could not only predict/decide tissue fate, but could also play part in the risk and degree of cerebral edema after reperfusion treatment.^22^, ^23^ Therefore, the slow progressors are less vulnerable toward time delay,^10^ explaining the deceleration of benefit loss in the relatively late time window. In the post hoc analysis of DEFUSE 2, where all the patients were with perfusion‐diffusion mismatch, no time modifying effect was discovered, which further implicates the potential role of collateral flow.^9^ Noteworthily, since all the patients in the aforementioned studies were punctured within 12 h from LKN, studies remain scarce for patients presenting in the later time window. The post hoc analysis of DEFUSE 3, using the same imaging selection criteria with DEFUSE 2 but with a more extended time windo, showed that longer time from imaging to puncture independently predicted poor outcomes, and patients with poor outcomes had impaired collateral flow in the univariate analysis.^21^ The speed of infarct growth closely depends on the level of collateral flow, which may collapse or fluctuate unpredictably.^11^, ^24^ Therefore, it is reasonable to deduce that the modifying effect of time on reperfusion still exists in EVT patients within 24 hours of LKN. Additionally, the selection criteria in our center roughly followed a mismatch ratio >1.2, leading to a selection of patients whose collateral flow were not as robust as patients in the DEFUSE serious studies with a mismatch ratio >1.8. Hence, the interaction between reperfusion index and different time metrics of the probability of 3‐month mRS 0–2 and infarct growth of our study is within expectation and can be explained by the influence of possible collateral failure over time.

This is also one of the few studies comparing the predictive performance of different reperfusion measurements obtained by repeated perfusion imaging and DSA on functional and imaging outcomes. In our study, reperfusion index≥0.9 was a better predictor of 3‐month functional dependency and infarct growth compared with reperfusion index≥0.5, mTICI≥2b and mTICI≥2c. Similar to our study using DT >3 s, a degree of 90% reperfusion measured by Tmax>6 s was suggested as an appropriate reperfusion threshold in outcome prediction, outplaying mTICI score.^16^ Recently, mTICI2c, defined as near complete perfusion except slow flow or few distal cortical emboli^15^or 90%–99% filling of the occluded vascular territory,^15^, ^25^ has been considered a more appropriate goal of EVT reperfusion than the traditional mTICI 2b, since it has a better performance in outcome prediction.^15^ However, in our study, though not statistically significant, mTICI≥2b numerically outplayed mTICI≥2c in predicting infarct growth and 3‐month mRS 0–2. These may be due to the increased number of passes and elongated procedure time in EVT in order to reach mTICI≥2c, and the negative effect of delayed reperfusion time outweighed the benefit of complete reperfusion. Our study demonstrated that reperfusion index had better predictive performance than mTICI. Since repeated CTP was conducted 24–28 hours after EVT in our study, the superiority of reperfusion index can be explained by the following reasons: First, distal embolism and the “no re‐flow” phenomenon, where incomplete capillary reperfusion with large artery recanalization occurred, can contribute to the sustaining hypoperfusion even after successful EVT. Mechanisms of the “no‐reflow” phenomenon include structural changes, such as red blood cell entrapment and activated polymorphonuclear leukocytes, as well as capillary flow pattern disruptions, such as dynamic flow stalls.^26^ Although the definition of hypoperfusion threshold varies between different studies, incomplete reperfusion after complete recanalization can lead to infarct growth and poor functional outcomes.^27^, ^28^, ^29^, ^30^ Second, the recanalized vessels could be re‐occluded due to new embolism or in‐situ thrombosis. Studies have shown that roughly 2% to 6% of successfully reperfused patients can suffer from artery re‐occlusion,^31^, ^32^ which can be detected by 24‐ to 48‐hour follow‐up CTP scan. The advantage of predictive ability of reperfusion index on outcomes over mTICI scores can also help to explain why the latter one had no statistically significant interaction with treatment time metrics on 3‐month mRS 0–2 and infarct growth. To be noted, most of the previous studies conducted post‐EVT perfusion scan shortly after EVT (mostly within 2 h), which allowed earlier identification of patients with hypoperfusion and timely intervention.^27^, ^33^, ^34^ Though our 24‐ to 48‐hour post‐EVT scan is relatively late and may lead to loss of the timing of early intervention to twist clinical deterioration, perfusion scan with contrast injection right after EVT may lead to radiation overdose and possible kidney injury. Anyway, our results suggest that for every eligible patient with EVT, a post‐EVT perfusion scan is encouraged to re‐assess their perfusion condition.

There are some inevitable limitations of this study. First, the sample size is very limited and half of the EVT patients were excluded due to lack of clinical or imaging data. Most of the excluded patients were unable to get 24‐ to 48‐hour CTP scan because of very severe clinical condition such as intubation and unstable vital signs. However, those patients were usually the fast‐progressors who were more vulnerable to treatment time delay. Therefore, the interaction between time metrics and outcomes might be more significant if these patients had been included. Additionally, the multiple comparisons of the AUCs of different reperfusion measurements were not Bonferroni adjusted due to the very limited sample size. Second, other reperfusion thresholds were not tested for their performance in outcome prediction. This is because the thresholds of reperfusion index≥0.5, reperfusion index≥0.9, mTICI≥2b, and mTICI≥2c are the most commonly used measurements in scientific studies and clinical practice. Third, reperfusion index was measured with decreased volume of DT >3 s rather than the more commonly used Tmax>6 s. However, studies have shown DT can measure acute ischemic lesion as accurate as Tmax.^35^ Lastly, some of infarct volumes were measured with NCCT rather than MRI. Therefore, imaging modality was adjusted the linear regression model of infarct growth prediction.

## 5 CONCLUSION

Reperfusion index and different treatment time had interactions on 3‐month mRS 0–2 and infarct growth in patients receiving EVT with 24‐hour of LKN. Reperfusion index≥0.9 might serve as a better proxy of good outcomes compared with other reperfusion measurements.

## ACKNOWLEGMENTS

This study was funded by the National Key R&D Program of China (2017YFC1308201), National Natural Science Foundation of China (82271352), Clinical Research Plan of SHDC (SHDC2020CR1041B), Shanghai Municipal Committee of Science and Technology (20Z11900802, 2018SHZDZX01), ZJLab (N/A).

## AUTHOR CONTRIBUTIONS

All author participated in data collection, critical review and revision of this manuscript. XC, QD designed the study. LH, YL analyzed the data. LH drafted this manuscript, prepared tables and figures.

## CONFLICT OF INTEREST

The authors have nothing to disclose.

## Supporting information


Tables S1‐S3.

## Data Availability

The data that support the findings of this study are available from the corresponding author upon reasonable request.

## References

[1] Powers WJ , Rabinstein AA , Ackerson T , et al. Guidelines for the early Management of Patients with Acute Ischemic Stroke: 2019 update to the 2018 guidelines for the early Management of Acute Ischemic Stroke: a guideline for healthcare professionals from the American Heart Association/American Stroke Association. Stroke. 2019;50(12):e344‐e418. 31662037 10.1161/STR.0000000000000211

[2] Jovin TG , Nogueira RG , Lansberg MG , et al. Thrombectomy for anterior circulation stroke beyond 6 h from time last known well (AURORA): a systematic review and individual patient data meta‐analysis. The Lancet. 2022;399(10321):249‐258. 10.1016/S0140-6736(21)01341-6 34774198

[3] Jahan R , Saver JL , Schwamm LH , et al. Association between time to treatment with endovascular reperfusion therapy and outcomes in patients with acute ischemic stroke treated in clinical practice. JAMA. 2019;322(3):252‐263. 31310296 10.1001/jama.2019.8286 PMC6635908

[4] Bourcier R , Goyal M , Liebeskind DS , et al. Association of time from stroke onset to groin puncture with quality of reperfusion after mechanical thrombectomy: a meta‐analysis of individual patient data from 7 randomized clinical trials. JAMA Neurol. 2019;76(4):405‐411. 30667465 10.1001/jamaneurol.2018.4510 PMC6459219

[5] Mazighi M , Chaudhry SA , Ribo M , et al. Impact of onset‐to‐reperfusion time on stroke mortality: a collaborative pooled analysis. Circulation. 2013;127(19):1980‐1985. 23671178 10.1161/CIRCULATIONAHA.112.000311

[6] Sheth SA , Jahan R , Gralla J , et al. Time to endovascular reperfusion and degree of disability in acute stroke. Ann Neurol. 2015;78(4):584‐593. 26153450 10.1002/ana.24474 PMC4955570

[7] Saver JL , Goyal M , van der Lugt A , et al. Time to treatment with endovascular thrombectomy and outcomes from ischemic stroke: a meta‐analysis. JAMA. 2016;316(12):1279‐1288. 27673305 10.1001/jama.2016.13647

[8] Prabhakaran S , Castonguay AC , Gupta R , et al. Complete reperfusion mitigates influence of treatment time on outcomes after acute stroke. J Neurointerv Surg. 2017;9(4):366‐369. 27073195 10.1136/neurintsurg-2016-012288

[9] Lansberg MG , Cereda CW , Mlynash M , et al. Response to endovascular reperfusion is not time‐dependent in patients with salvageable tissue. Neurology. 2015;85(8):708‐714. 26224727 10.1212/WNL.0000000000001853 PMC4553034

[10] Hwang YH , Kang DH , Kim YW , Kim YS , Park SP , Liebeskind DS . Impact of time‐to‐reperfusion on outcome in patients with poor collaterals. AJNR Am J Neuroradiol. 2015;36(3):495‐500. 25376808 10.3174/ajnr.A4151 PMC8013039

[11] Campbell BC , Christensen S , Tress BM , et al. Failure of collateral blood flow is associated with infarct growth in ischemic stroke. J Cereb Blood Flow Metab. 2013;33(8):1168‐1172. 23652626 10.1038/jcbfm.2013.77 PMC3734777

[12] Lansberg MG , Straka M , Kemp S , et al. MRI profile and response to endovascular reperfusion after stroke (DEFUSE 2): a prospective cohort study. The Lancet Neurology. 2012;11(10):860‐867. 22954705 10.1016/S1474-4422(12)70203-X PMC4074206

[13] Albers GW , Marks MP , Kemp S , et al. Thrombectomy for stroke at 6 to 16 hours with selection by perfusion imaging. N Engl J Med. 2018;378(8):708‐718. 29364767 10.1056/NEJMoa1713973 PMC6590673

[14] Zaidat OO , Yoo AJ , Khatri P , et al. Recommendations on angiographic revascularization grading standards for acute ischemic stroke: a consensus statement. Stroke. 2013;44(9):2650‐2663. 23920012 10.1161/STROKEAHA.113.001972 PMC4160883

[15] Tung EL , McTaggart RA , Baird GL , et al. Rethinking thrombolysis in cerebral infarction 2b: which thrombolysis in cerebral infarction scales best define near complete recanalization in the modern thrombectomy era? Stroke. 2017;48(9):2488‐2493. 28775136 10.1161/STROKEAHA.117.017182

[16] Tan Z , Parsons M , Bivard A , et al. Optimal tissue reperfusion estimation by computed tomography perfusion post‐thrombectomy in acute ischemic stroke. Stroke. 2021;52(12):e760‐e763. 34670411 10.1161/STROKEAHA.121.034581

[17] Lin L , Bivard A , Krishnamurthy V , Levi CR , Parsons MW . Whole‐brain CT perfusion to quantify acute ischemic penumbra and core. Radiology. 2016;279(3):876‐887. 26785041 10.1148/radiol.2015150319

[18] Saver JL , Goyal M , Bonafe A , et al. Stent‐retriever thrombectomy after intravenous t‐PA vs. t‐PA alone in stroke. N Engl J Med. 2015;372(24):2285‐2295. 25882376 10.1056/NEJMoa1415061

[19] Janes H , Longton G , Pepe M . Accommodating covariates in ROC analysis. Stata J. 2009;9(1):17‐39. 20046933 PMC2758790

[20] Pepe M , Longton G , Janes H . Estimation and comparison of receiver operating characteristic curves. Stata J. 2009;9(1):1‐16. 20161343 PMC2774909

[21] Heit JJ , Mlynash M , Christensen S , et al. What predicts poor outcome after successful thrombectomy in late time windows? J Neurointerv Surg. 2021;13(5):421‐425. 32554693 10.1136/neurintsurg-2020-016125

[22] Klug J , Dirren E , Preti MG , et al. Integrating regional perfusion CT information to improve prediction of infarction after stroke. J Cereb Blood Flow Metab. 2021;41(3):502‐510. 32501132 10.1177/0271678X20924549 PMC7922756

[23] Faizy TD , Kabiri R , Christensen S , et al. Perfusion imaging‐based tissue‐level collaterals predict ischemic lesion net water uptake in patients with acute ischemic stroke and large vessel occlusion. J Cereb Blood Flow Metab. 2021;41(8):2067‐2075. 33557694 10.1177/0271678X21992200 PMC8327120

[24] Lin L , Yang J , Chen C , et al. Association of collateral status and ischemic core growth in patients with acute ischemic stroke. Neurology. 2021;96(2):e161‐e170. 33262233 10.1212/WNL.0000000000011258

[25] Liebeskind DS , Bracard S , Guillemin F , et al. eTICI reperfusion: defining success in endovascular stroke therapy. J Neurointerv Surg. 2019;11(5):433‐438. 30194109 10.1136/neurintsurg-2018-014127

[26] Erdener SE , Tang J , Kilic K , et al. Dynamic capillary stalls in reperfused ischemic penumbra contribute to injury: a hyperacute role for neutrophils in persistent traffic jams. J Cereb Blood Flow Metab. 2021;41(2):236‐252. 32237951 10.1177/0271678X20914179 PMC8370003

[27] Kosior JC , Buck B , Wannamaker R , et al. Exploring reperfusion following endovascular Thrombectomy. Stroke. 2019;50(9):2389‐2395. 31366315 10.1161/STROKEAHA.119.025537

[28] Haussen DC , Nogueira RG , Elhammady MS , et al. Infarct growth despite full reperfusion in endovascular therapy for acute ischemic stroke. J Neurointerv Surg. 2016;8(2):117‐121. 25540178 10.1136/neurintsurg-2014-011497

[29] Ter Schiphorst A , Charron S , Hassen WB , et al. Tissue no‐reflow despite full recanalization following thrombectomy for anterior circulation stroke with proximal occlusion: a clinical study. J Cereb Blood Flow Metab. 2021;41(2):253‐266. 32960688 10.1177/0271678X20954929 PMC8370008

[30] Ng FC , Churilov L , Yassi N , et al. Prevalence and significance of impaired microvascular tissue reperfusion despite macrovascular angiographic reperfusion (No‐reflow). Neurology. 2022;98(8):e790‐e801. 34906976 10.1212/WNL.0000000000013210

[31] Marto JP , Strambo D , Hajdu SD , et al. Twenty‐four‐hour reocclusion after successful mechanical Thrombectomy: associated factors and long‐term prognosis. Stroke. 2019;50(10):2960‐2963. 31535931 10.1161/STROKEAHA.119.026228

[32] Mosimann PJ , Kaesmacher J , Gautschi D , et al. Predictors of unexpected early Reocclusion after successful mechanical thrombectomy in acute ischemic stroke patients. Stroke. 2018;49(11):2643‐2651. 30355192 10.1161/STROKEAHA.118.021685

[33] Rubiera M , Garcia‐Tornel A , Olive‐Gadea M , et al. Computed tomography perfusion after thrombectomy: an immediate surrogate marker of outcome after recanalization in acute stroke. Stroke. 2020;51(6):1736‐1742. 32404034 10.1161/STROKEAHA.120.029212

[34] Shin J , Kim YS , Jang HS , et al. Perfusion recovery on TTP maps after endovascular stroke treatment might predict favorable neurological outcomes. Eur Radiol. 2020;30(12):6421‐6431. 32676783 10.1007/s00330-020-07066-3

[35] Lin L , Bivard A , Kleinig T , et al. Correction for delay and dispersion results in more accurate cerebral blood flow ischemic Core measurement in acute stroke. Stroke. 2018;49(4):924‐930. 29500248 10.1161/STROKEAHA.117.019562

