# Supplementary material for: Reperfusion measurements, treatment time, and outcomes in patients receiving endovascular treatment within 24 hours of last known well
Source: CNS Neurosci Ther. 2023 Jan 4;29(4):1067–74. doi: 10.1111/cns.14080 (PMC10018078; doi:10.1111/cns.14080)
Supplement: Supplementary file 1 — Tables S1‐S3. [file CNS-29-1067-s001.docx]

**Supplementary Table 1** Baseline and follow-up clinical and imaging data of patients with 3-month mRS 0-2 and patients with 3-month mRS 3-6^†^

|  | mRS 0-2 (n=34) | mRS 3-6 (n=39) | P |
| --- | --- | --- | --- |
| Baseline Demographic Data |  |  |  |
| **Age,** median (IQR), yrs | 64.5 (51.8, 72.3) | 71.0 (60.0, 80.0) | 0.01 |
| **Male** | 21 (61.8%) | 23 (59.0%) | 0.81 |
| **Baseline Glucose,** median (IQR)**,** mmol/L | 6.9 (6.3, 7.6) | 7.3 (6.0, 8.6) | 0.34 |
| **Baseline SBP,** mean (SD), mmHg | 137.4 (4.5) | 146.7 (3.1) | 0.09 |
| **Baseline DBP,** mean (SD), mmHg | 81.4 (2.1) | 86.4 (2.1) | 0.10 |
| **Baseline NIHSS,** mean (SD) | 13.9 (0.9) | 13.6 (0.8) | 0.82 |
| **Medical History** |  |  |  |
| History of Smoking | 15 (44.1%) | 12 (30.8%) | 0.22 |
| History of Hypertension | 15 (44.1%) | 24 (61.5%) | 0.14 |
| History of Atrial Fibrillation | 10 (29.4%) | 12 (30.8%) | 0.90 |
| History of Dyslipidemia | 3 (8.8%) | 6 (15.4%) | 0.49 |
| History of Diabetes Mellitus | 7 (20.6%) | 7 (18.0%) | 0.78 |
| Past History of Stroke | 7 (20.6%) | 7 (18.0%) | 0.78 |
| History of Congestive Heart Failure | 0 (0.0%) | 1 (2.6%) | 0.53 |
| History of Ischemic Heart Disease | 2 (5.9%) | 2 (5.1%） | 0.64 |
| Taking Antiplatelet Prior to Stroke | 5 (14.7%) | 8 (20.5%) | 0.52 |
| Taking Anticoagulant Prior to Stroke | 6 (17.7%) | 2 (5.1%) | 0.14 |
| **Cause of Stroke** |  |  | 0.39 |
| Large Artery Atherosclerosis | 18 (52.9%) | 19 (48.7%) |  |
| Cardiac Embolism | 13 (38.3%) | 12 (30.8%) |  |
| Others^‡^ | 3 (8.8%) | 8 (20.5%) |  |
| **Occlusion Site** |  |  | 0.47 |
| ICA | 7 (20.6%) | 10 (25.6%) |  |
| MCA-M1 | 20 (58.8%) | 25 (64.1%) |  |
| MCA-M2 | 3 (8.8%) | 3 (7.7%) |  |
| Tandem | 4 (11.8%) | 1 (2.6%) |  |
| **Reperfusion Treatment Procedure** |  |  |  |
| **Procedure Time** |  |  |  |
| Time from LKN to ER arrival, median (IQR), min | 159.5 (97.3, 353.0) | 201.0 (78.0, 307.0) | 0.69 |
| Time from LKN to acute multimodal imaging, median (IQR), min | 223.0 (122.5, 419.8) | 233.0 (118.0, 359.0) | 0.94 |
| Time from LKN to groin puncture, median (IQR), min | 345.0 (203.8, 582.3) | 350.0 (235.0, 512.0) | 0.78 |
| Time from LKN to reperfusion, median (IQR), min^§^ | 435.0 (345.0, 651.3) | 409.0 (290.0, 540.0) | 0.71 |
| **Intravenous thrombolysis** | 16 (47.1%) | 14 (35.9%) | 0.33 |
| **Final mTICI** |  |  | 0.06 |
| 0 | 1 (2.9%) | 9 (23.1%) |  |
| 1 | 1 (2.9%) | 3 (7.7%) |  |
| 2a | 3 (8.8%) | 5 (12.8%) |  |
| 2b | 9 (26.5%) | 6 (15.4%) |  |
| 2c | 2 (5.9%) | 4 (10.3%) |  |
| 3 | 18 (52.9%) | 12 (30.8%) |  |
| mTICI≥2b | 29 (85.3%) | 22 (56.4%） | 0.01 |
| mTICI≥2c | 20 (58.8%) | 16 (41.3%) | 0.13 |
| **Imaging data** |  |  |  |
| Baseline infarct core, median (IQR), mL | 8.5 (3.0, 17.0) | 10.0 (4.0, 30.0) | 0.60 |
| Baseline DT>3s, median (IQR), mL | 78.0 (39.3, 119.5) | 94.0 (60.0, 157.0) | 0.15 |
| Reperfusion index, median (IQR) | 1.0 (0.9, 1.0) | 0.8 (0.2,1.0) | 0.001 |
| Reperfusion index ≥0.5 | 32 (94.1%) | 26 (66.7%) | 0.004 |
| Reperfusion index ≥0.9 | 25 (73.5%) | 15 (38.5%) | 0.003 |
| **Outcome** |  |  |  |
| Infarct growth, median (IQR), mL^¶^ | 7.4 (-6.6, 22.4) | 43.9 (8.8, 124.5) | 0.001 |
| ^†^ Data are presented as number (percentage) of patients unless otherwise indicated.  ^‡^ Other causes of stroke included embolic stroke of undetermined source, hypercoagulation, stroke of undermined causes, dissection of ipsilateral carotid artery, syphilis and hypoperfusion.  ^§^ Only calculated for patients who had final mTICI≥2a, n=59  ^¶^ Final infarct volume of 18 Patients were measured using NCCT.  Abbreviations: IQR Interquartile range; SD Standard deviation; SBP systolic blood pressure; DBP diastolic blood pressure; NIHSS National Institutes of Health Stroke Scale; TIA transient ischemic attack; ICA internal carotid artery; MCA-M1 M1 segment of middle cerebral artery; MCA-M2 M2 segment of middle cerebral artery; LKN Last known well; ER Emergency room; mTICI modified Treatment in Cerebral Ischemia; DT Delay time; mRS modified Rankin Scale; NCCT Non-contrast computed tomography | | | |

**Supplementary Table 2** Univariate analysis of the correlation between baseline data and follow-up clinical and imaging data and infarct growth^†^

|  | | P |
| --- | --- | --- |
| Baseline Demographic Data | |  |
| **Age,** yrs | | 0.18 |
| **Male** | | 0.72 |
| **Baseline Glucose,** mmol/L | | 0.70 |
| **Baseline SBP,** mmHg | | 0.79 |
| **Baseline DBP,** mmHg | | 0.72 |
| **Baseline NIHSS** | | 0.10 |
| **Medical History** | |  |
| History of Smoking | | 0.27 |
| History of Hypertension | | 0.22 |
| History of Atrial Fibrillation | | 0.99 |
| History of Dyslipidemia | | 0.29 |
| History of Diabetes Mellitus | | 0.06 |
| Past History of Stroke | | 0.71 |
| History of Congestive Heart Failure | | 0.37 |
| History of Ischemic Heart Disease | | 0.28 |
| Taking Antiplatelet Prior to Stroke | | 0.93 |
| Taking Anticoagulant Prior to Stroke | | 0.43 |
| **Cause of stroke** | | 0.19 |
| **Occlusion Site** | | 0.79 |
| **Reperfusion Treatment Procedure** | |  |
| **Procedure Time** | |  |
| Time from LKN to ER arrival, min | | 0.77 |
| Time from LKN to acute multimodal imaging, min | | 0.70 |
| Time from LKN to groin puncture, min | | 0.66 |
| Time from LKN to reperfusion, min^‡^ | | 0.87 |
| **Intravenous thrombolysis** | | 0.54 |
| **Final mTICI** | 0.16 | |
| mTICI≥2b | | 0.23 |
| mTICI≥2c | | 0.17 |
| **Imaging data** | |  |
| Baseline infarct core, mL | | 0.05 |
| Baseline DT>3s, mL | | 0.53 |
| Reperfusion index | | 0.001 |
| Reperfusion index ≥0.5 | | 0.02 |
| Reperfusion index ≥0.9 | | 0.003 |
| **Outcome** | |  |
| 3-month mRS 0-2 | | 0.001 |
| ^†^ Final infarct volume of 18 Patients were measured using NCCT.  ^‡^ Only calculated for patients who had final mTICI≥2a, n=59  Abbreviations: IQR Interquartile range; SD Standard deviation; SBP systolic blood pressure; DBP diastolic blood pressure; NIHSS National Institutes of Health Stroke Scale; TIA transient ischemic attack; LKN Last known well; ER Emergency room; mTICI modified Treatment in Cerebral Ischemia; DT Delay time; mRS modified Rankin Scale; NCCT Non-contrast computed tomography | | |

| **Supplementary Table 3** Interaction between reperfusion index, mTICI score and different time metrics on outcomes | |
| --- | --- |
|  | P for Interaction |
| **mRS 0-2**^†^ |  |
| Reperfusion index |  |
| Time from LKN to ER arrival | 0.07 |
| Time from LKN to reperfusion (n=59) | 0.18 |
| mTICI |  |
| Time from LKN to ER arrival | 0.27 |
| Time from LKN to acute multimodal imaging | 0.21 |
| Time from LKN to groin puncture | 0.20 |
| Time from LKN to reperfusion (n=59) | 0.44 |
| **Infarct growth**^‡^ |  |
| Reperfusion index |  |
| Time from LKN to acute multimodal imaging | 0.64 |
| mTICI |  |
| Time from LKN to ER arrival | 0.54 |
| Time from LKN to acute multimodal imaging | 0.21 |
| Time from LKN to groin puncture | 0.81 |
| Time from LKN to reperfusion (n=59) | 0.20 |
| ^†^ Multivariate-adjusted for age and baseline NIHSS  ^‡^ Multivariate-adjusted for a history of diabetes mellitus, baseline core volume and MR/NCCT scan  Abbreviations: mTICI modified treatment in cerebral ischemia ; mRS modified Rankin Scale; LKN last known well; ER emergency room; NIHSS National Institutes of Health Stroke Scale; MR magnetic resonance; NCCT non-contrast computed tomography | |
